# Bitter Taste and Olfactory Receptors: Beyond Chemical Sensing in the Tongue and the Nose

**DOI:** 10.1007/s00232-021-00182-1

**Published:** 2021-06-25

**Authors:** Mercedes Alfonso-Prieto

**Affiliations:** 1 Institute for Advanced Simulations IAS‐5/Institute for Neuroscience and Medicine INM‐9, Computational Biomedicine, Forschungszentrum Jülich GmbH, Jülich, Germany; 2 Medical Faculty, Cécile and Oskar Vogt Institute for Brain Research, University Hospital Düsseldorf, Heinrich Heine University Düsseldorf, Düsseldorf, Germany

**Keywords:** Bitter taste, Olfaction, Chemosensory receptors, TAS2Rs, ORs, Odorant

## Abstract

**Abstract:**

The Up-and-Coming-Scientist section of the current issue of the Journal of Membrane Biology features the invited essay by Dr. Mercedes Alfonso-Prieto, Assistant Professor at the Forschungszentrum Jülich (FZJ), Germany, and the Heinrich-Heine University Düsseldorf, Vogt Institute for Brain Research. Dr. Alfonso-Prieto completed her doctoral degree in chemistry at the Barcelona Science Park, Spain, in 2009, pursued post-doctoral research in computational molecular sciences at Temple University, USA, and then, as a Marie Curie post-doctoral fellow at the University of Barcelona, worked on computations of enzyme reactions and modeling of photoswitchable ligands targeting neuronal receptors. In 2016, she joined the Institute for Advanced Science and the Institute for Computational Biomedicine at the FZJ, where she pursues research on modeling and simulation of chemical senses. The invited essay by Dr. Alfonso-Prieto discusses state-of-the-art modeling of molecular receptors involved in chemical sensing – the senses of taste and smell. These receptors, and computational methods to study them, are the focus of Dr. Alfonso-Prieto’s research. Recently, Dr. Alfonso-Prieto and colleagues have presented a new methodology to predict ligand binding poses for GPCRs, and extensive computations that deciphered the ligand selectivity determinants of bitter taste receptors. These developments inform our current understanding of how taste occurs at the molecular level.

**Graphic Abstract:**

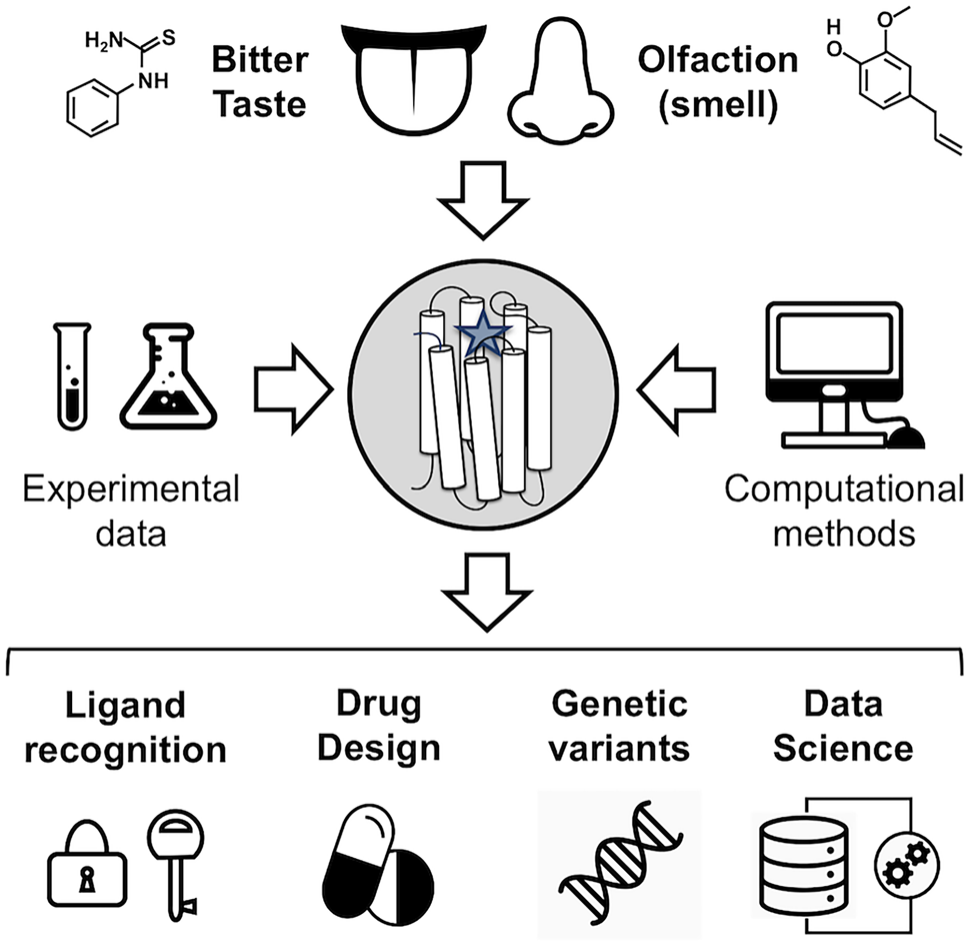


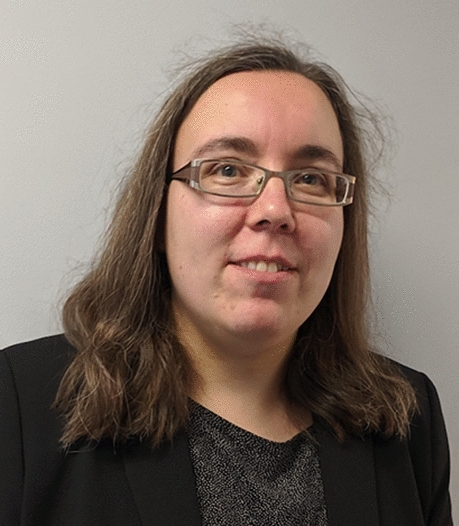


## Introduction

In contrast with vision or hearing, the senses of taste and smell (or olfaction) involve the detection of chemicals present in the environment and thus are considered as “chemical senses”. They inform about the aroma and flavor of food and beverages and act as warning system for toxic substances. Hence, these two chemical senses strongly affect human well-being, food acceptance and intake (Tepper et al. 2020; Boesveldt and Parma 2021), as well as drug compliance (Pawar and Kumar 2002; Menella et al. 2013), especially in children. In addition, taste and smell impact key brain processes (Sullivan et al. 2015; Boesveldt and de Graaf 2017; Sabiniewicz et al. 2021), such as memory, emotional responses or behavior.

Taste and olfaction impairments strongly affect the quality of life, social interactions and dietary habits (Mainland et al. 2020). Moreover, the loss of taste and smell is a common symptom of Parkinson’s and Alzheimer’s diseases (Robert et al. 2016; Tarakad and Jankovic 2017; Oppo et al. 2020) and has been recently shown to be one of the symptoms of COVID-19 infection (Parma et al. 2020; Gerkin et al. 2021; Pierron et al. 2020). Therefore, taste and olfaction are also clinically relevant.

The molecules responsible for taste and smell (tastants and odorants, respectively) are extremely chemically diverse (Malnic et al. 1999; Meyerhof et al. 2010). Consequently, the human genome contains a large number of membrane proteins dedicated to recognize them (Buck and Axel 1991; Adler et al. 2000; Chandrashekar et al. 2006). In particular, the two largest chemosensory families correspond to olfactory receptors (ORs) and taste 2 (or bitter taste) receptors (TAS2Rs). Although originally identified in nose and mouth, these receptors have been later shown to be expressed also in other parts of the body (Behrens and Meyerhof 2011; Massberg and Hatt 2018; Dalesio et al. 2018; Behrens and Meyerhof 2019). This extraoral and extranasal expression indicates that ORs and TAS2Rs can also play a role in other physiological and pathological processes, besides bitter taste and smell perception, and thus opens the way for new therapeutical interventions (Lee et al. 2019; Di Pizio et al. 2019).

**Fig. 1:**
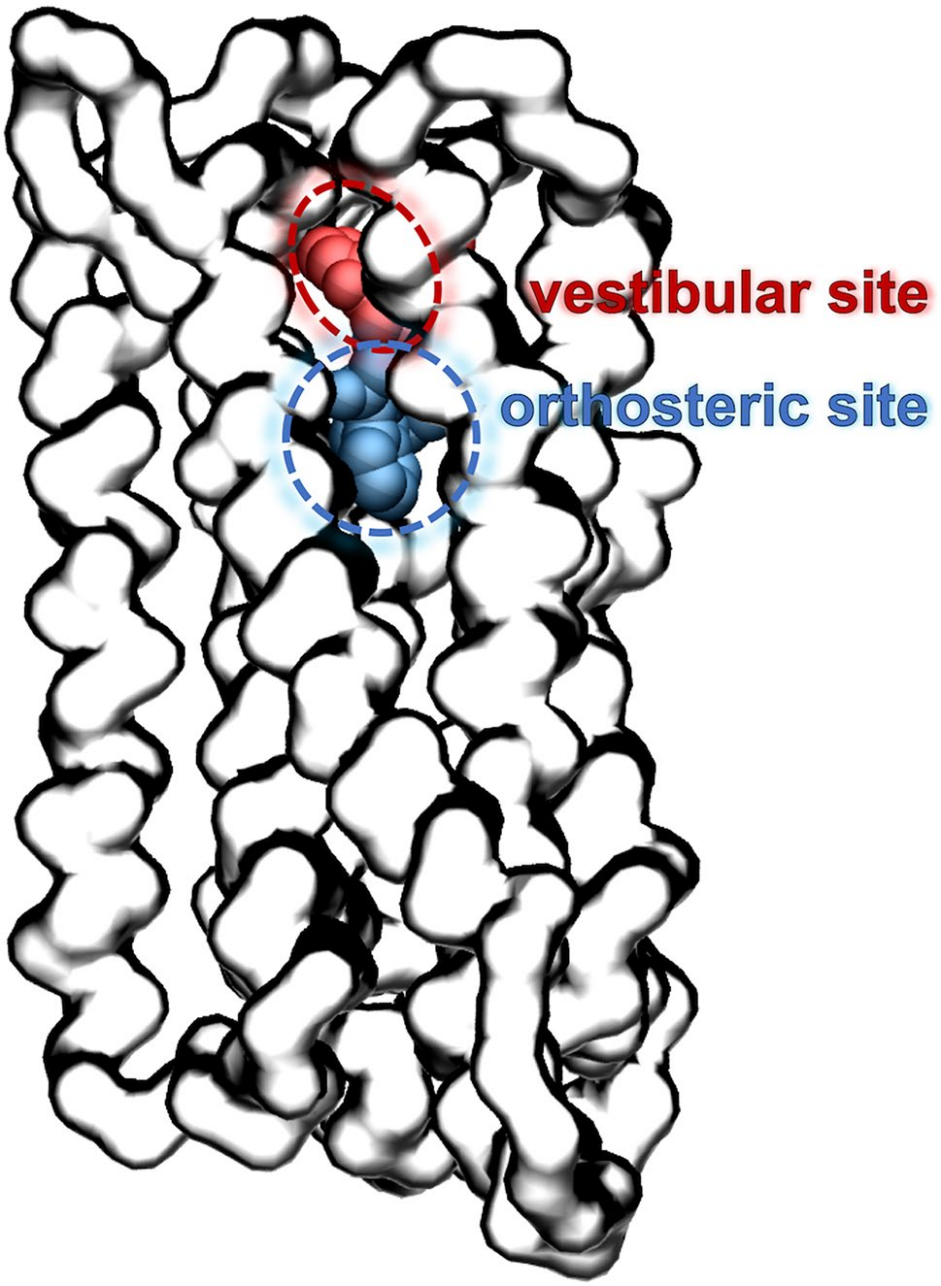
Computational model of TAS2R46 in complex with the bitter tastant strychnine, showing the two-site architecture explored in the simulations. The ligand pose in the vestibular site is colored in red, whereas the pose in the orthosteric site is in blue. Data were taken from reference (Sandal et al. 2015) (Color figure online)

Both TAS2Rs and ORs are G-protein coupled receptors (GPCRs). The GPCR superfamily is the largest in the human genome, with approx. 800 genes, of which half correspond to chemosensory receptors (Venter et al., 2001; Alexander et al., 2019). Within the class A-D system (Fredriksson et al. 2003; Schioth and Fredriksson 2005; Lagerstrom and Schioth 2008), ORs are part of class A, whereas the classification of TAS2Rs is still under debate (as either class A, or class F or a new class T) (Nordstrom et al., 2011; de March et al. 2015; Di Pizio et al. 2016; Munk et al., 2016a). Regardless of their classification, TAS2Rs and ORs share the same topology, with a seven transmembrane (TM) helix bundle (see Fig. 1). Bitter tastants and odorants are recognized by their corresponding receptor by binding in a cavity located in the extracellular part of the TM bundle. Ligand binding triggers a conformational change of the receptor that promotes binding of the associated G-protein in the intracellular part. This, in turn, activates the G-protein, which acts as a transducer, initiating an intracellular signaling cascade that results in a cellular response (Alexander et al., 2019).

ORs and TAS2Rs can identify a wide range of ligands and thus are considered promiscuous receptors. In humans, the approx. 400 ORs can recognize up to one trillion different odorants (Bushdid et al. 2014), whereas the 25 TAS2Rs detect around 1000 bitter tastants (Behrens and Meyerhof 2018; Dagan-Wiener et al. 2019). One receptor may be able to bind several molecules and the same ligand can be recognized by multiple receptors (Malnic et al., 1999; Krautwurst 2008; Meyerhof et al. 2010; Ji et al., 2014; Dunkel et al. 2014; Di Pizio and Niv 2015). This complex combinatorial code is still not fully understood, hindering identification of new ligands and receptor deorphanization.

The GPCR superfamily is highly pharmacologically relevant, with approx. 110 GPCRs being targeted by one third of all the FDA-approved drugs (Hauser et al. 2017, 2018). Similarly, ORs and TAS2Rs appear to be promising candidates for future drug design efforts (Lee et al. 2019; Di Pizio et al. 2019; Ahmad et al. 2020). Some examples include bronchodilators targeting TAS2Rs expressed in the airways (Nayak et al. 2019) or the sandalwood odorant used as hair loss therapy by stimulating OR2AT4 expressed in human hair follicles (Cheret 2018). Nonetheless, further therapeutical applications will require a more comprehensive characterization of the extranasal and extraoral roles of these receptors, as well as better understanding of the binding determinants of chemosensory receptors (Lee et al. 2019; Di Pizio et al. 2019; Ahmad et al. 2020).

Another common feature between GPCRs and ORs/TAS2Rs is the presence of genetic variants across the human population. In the case of GPCR drug targets, these variants can affect the pharmacological response of the receptor (Hauser et al. 2018). For chemosensory receptors, they have been mostly studied in the context of bitter taste/smell sensitivity, but they can also affect health status and propensity to certain diseases (Shaw et al. 2011; Mainland et al. 2014; Logan 2014; Chakraborty et al. 2019; Risso et al. 2021). One of the most well characterized examples is TAS2R38. After the serendipitous discovery of differences in the individual sensitivity to phenylthiocarbamide (PTC, a TAS2R38 ligand) (Fox 1932; Blakeslee 1932), a large-scale study (Blakeslee and Fox 1932) showed two main categories in the population, non-tasters and super-tasters. Later, a genome-wide linkage analysis (Kim et al. 2003) confirmed that sequence variants in the TAS2R38 gene have a direct influence in the observed PTC taste sensitivity. In addition to affecting food preferences (Robino et al. 2014), TAS2R38 polymorphisms have been recently shown to influence respiratory innate immunity mechanisms and susceptibility to chronic rhinosinusitis ((Jeruzal-Swiatecka et al. 2020) and references within).

## Computational Structural Methods

Understanding the molecular determinants of odorant or bitter tastant binding to their corresponding receptor requires structural information (Di Pizio and Niv 2014; de March et al. 2015; Fierro et al. 2017; Behrens et al. 2018a, b; Alfonso-Prieto et al. 2019a, b). X-ray or cryo-EM structures have been solved for only ~ 10% of human GPCRs (Munk et al. 2019; Bender et al. 2020; Kooistra et al. 2021) and, in particular, experimental structures of human ORs and TAS2Rs are still missing (https:// gpcrdb.org/structure/statistics; version 2021-01-27). Hence, computational methods have been used to fill the structural gap for chemosensory receptors, in particular homology modeling, molecular docking and molecular dynamics (MD) simulations (Di Pizio and Niv 2014; de March et al. 2015; Behrens et al. 2018a, b; Alfonso-Prieto et al. 2019a, b).

Building a high-resolution receptor homology model requires the identification of a template with sequence identity above 35% (Chothia and Lesk, 1986; Olivella et al. 2013; Piccoli et al. 2013). This is often not straightforward for the large and heterogeneous GPCR superfamily: only 10% of the GPCRs of unknown experimental structure have a closely related template with sequence identity above the 35% threshold (Zhang et al. 2006; Bender et al. 2020). In the case of ORs and TAS2Rs, their sequence identity with any of the available templates is unfortunately lower (< 20%) and thus the resulting homology models are low resolution (Fierro et al. 2017). Nonetheless, several computational approaches have been proposed to overcome this limitation, including homology modeling based on multiple templates or generation of an ensemble of models (Biarnés et al. 2010; de March et al. 2015; Di Pizio et al. 2017; Dagan-Wiener et al. 2019; Nowak et al. 2018; Bushdid et al. 2019; Spaggiari et al. 2020).

Molecular docking is then used in combination with these homology models to predict the binding mode of known ligands and, in some cases, identify new compounds. However, the accuracy of the docking results is limited by the low quality of the initial receptor models. In particular, the receptor-ligand interactions depend on the orientation of the amino acid side chains, which is uncertain in such low resolution models (Fierro et al. 2017). Hence, strategies aiming at enhancing the sampling of the conformational space of the receptor-ligand complex have been applied to overcome this limitation. These include flexible docking approaches and experimental data-driven model refinement (Di Pizio et al. 2017; Nowak et al. 2018; Bushdid et al. 2018; Bushdid et al. 2019; Di Pizio et al. 2020a, b), as well as molecular dynamics (MD) simulations (Gelis et al. 2012; Charlier et al. 2012; Charlier et al. 2013; Topin et al. 2014; Marchiori et al. 2013; Sandal et al. 2015; Li et al. 2016; Fierro et al. 2017; Ahmed et al. 2018; Fierro et al. 2019; Alfonso-Prieto et al. 2019a, 2019b; Haag et al., 2020; Schneider et al. 2020).

The thus-generated receptor-ligand complex models are validated by comparison with experimental data. Site-directed mutagenesis combined with functional experiments can be used to verify the predicted receptor binding residues (Munk et al. 2016b). In addition, the predicted ligand binding mode(s) can be compared against structure–activity relationship data (Vaas et al. 2018). Therefore, the interplay between computational and experimental data is crucial to obtain an accurate molecular picture of bitter taste and olfaction (Behrens et al. 2018a, b; Alfonso-Prieto et al. 2019a; Spaggiari et al. 2020).

The above described computational molecular modeling approaches have offered crucial insights into the ligand promiscuity of chemosensory receptors. Both TAS2Rs and ORs can recognize a broad range of ligands, yet they are selective. Based on mutagenesis data on TAS2Rs, it was proposed that such discriminating promiscuity could be achieved by a so-called “access control” that is able to dismiss the wrong compounds (Brockhoff et al. 2010). The molecular basis of such mechanism has been revealed using MD. Simulations showed that bitter tastants or odorants can explore not only one but two binding pockets in the corresponding chemosensory receptor (Sandal et al. 2015; Bushdid et al. 2019). This two-site architecture (Fig. 1) acts as a two-step verification system: the vestibular site (located close to the extracellular loops) filters out the receptor cognate ligands, which can then move downwards to the orthosteric site (situated inside the seven TM helix bundle), in order to trigger receptor activation. The interplay of the two binding sites is further validated by the observation that mutations of residues identified in either site affect the receptor response to its ligands. Moreover, the position of the orthosteric site in TAS2Rs and ORs coincides with that observed in experimental structures of other class A GPCRs in complex with their ligands (Venkatakrishnan et al. 2013; Latorraca et al. 2017). Similarly, the vestibular site overlaps with the extracellular allosteric site of other class A GPCRs (Thal et al. 2018; Latorraca et al. 2017). Further computational and experimental studies on other chemosensory receptors are needed to confirm whether this two-site architecture is conserved across the TAS2R and OR families.

Several FDA-approved drugs taste bitter (Dagan-Wiener et al. 2017; Di Pizio et al. 2019) and others affect olfactory perception (Lötsch et al. 2012). Moreover, TAS2Rs and ORs are also expressed in other parts of the body outside of the tongue and the nose, respectively, where they are involved in (yet not fully characterized) physiological and pathological processes (Lee et al. 2019; Di Pizio et al. 2019; Ahmad et al. 2020). Taken together, this suggests that bitter taste and odor molecules may be potential drug candidates (Di Pizio et al. 2019). A few computational molecular modeling studies have been carried out to explore the pharmacological potential of TAS2Rs and ORs (Tong et al. 2017; Nowak et al. 2018; Di Pizio et al. 2020a, b). Besides generating structural models of the receptor/ligand pairs already known (Levit et al. 2014; Tong et al. 2017; Nowak et al. 2018), computational approaches can also be used to design chemical modifications to improve ligand-receptor affinity and other drug-like properties (Di Pizio et al. 2020a, b). For instance, TAS2R14 has been recently studied as potential drug target against respiratory infections (Di Pizio et al. 2020a, b) due to its association with innate immune responses (Hariri et al. 2017) and its ability to bind clinical drugs that taste bitter, such as flufenamic acid (Levit et al. 2014; Behrens et al. 2018a, b). By integrating experimental mutagenesis data, homology modeling and molecular docking, an initial structural model of TAS2R14 in complex with flufenamic acid was generated (Levit et al. 2014; Nowak et al. 2018). Then, a combinatorial library of flufenamic acid derivatives was virtually screened against this model and the best candidate compounds were selected based on their docking score and visual inspection. These ligands, as well as additional analogs designed using medicinal chemistry concepts, were synthesized and subsequently tested with in vitro functional assays, resulting in the identification of new TAS2R14 agonists with nanomolar potency. Moreover, these experimental data were further used to refine the initial TAS2R14 model and obtain a better molecular description of the ligand binding modes (Di Pizio et al. 2020a, b). Altogether, this success story shows the potential of integrated experimental-computational approaches for ligand design for TAS2Rs and eventually ORs and opens the way to exploit the largely untapped pharmacological potential of these chemosensory receptors.

In addition, computational molecular modeling has been useful to understand the effect of genetic variants of TASR2s and ORs on ligand sensitivity (Biarnés et al. 2010; Marchiori et al. 2013; Geithe et al. 2017; March et al., 2015; Cierco-Jiménez et al. 2021). In the aforementioned case of TAS2R38, an ensemble of receptor homology models was generated, followed by molecular docking of PTC and further refinement with multiscale MD simulations (Biarnés et al. 2010; Marchiori et al. 2013). The resulting TAS2R38/PTC complex models allowed the identification of the residues putatively involved in binding, which were subsequently validated using mutagenesis and functional assays. Moreover, such models showed that amino acid 296, which varies between super-taster and non-taster variants, is not involved in ligand binding. Instead, residue 296 (located in TM7 at position 7.52 in the Ballesteros and Weinstein (1995) numbering scheme) faces F255 in the adjacent TM6, a helix which moves significantly during GPCR activation (Tehan et al. 2014; Venkatakrishnan et al. 2016; Weis and Kobilka 2018; Filipek 2019). Therefore, the interaction between these residues 296 and 255 (or lack thereof) can have an effect on receptor activation (Biarnés et al. 2010). The TAS2R38 variant present in the super-taster haplotype contains Val at position 296 and thus a hydrophobic interaction V296-F255 can be formed. This correlates with the in vitro activity measurements and the higher PTC sensitivity phenotype. On the contrary, the TAS2R38 variant present in the non-taster haplotypes contains Ile at position 296, a bulkier amino acid that will disrupt such interaction. This is in agreement with the lack of or reduced activity of the Ile296-containing TAS2R38 mutants and the lower PTC sensitivity phenotype. This hypothesis was further confirmed by creating a double swap-mutant F255V/V296F, which shows normal activity, consistent with the recovery of the proposed interaction (Biarnés et al. 2010). Interestingly, a subsequent bioinformatics analysis showed that, besides Val296 in TAS2R38, a hydrophobic residue at the equivalent position 7.52 is conserved for most TAS2Rs, as well as class A GPCRs. Additional comparisons against active-inactive pairs of experimental structures for class A GPCRs, together with mutagenesis data, further suggest that this position might be a not-yet-characterized activation microswitch for class A GPCRs (Fierro et al. 2017).

## Data Science Approaches

Given the wide chemical space covered by odorants and bitter tastants, as well as the large number of ORs and TAS2Rs, it is not surprising that several online resources have been developed to compile the vast amount of data associated to these chemosensory receptors. Moreover, the combinatorial nature of bitter taste and olfaction is perfectly suited for the application of data-driven approaches, in particular machine learning (Lötsch et al., 2019).

For TAS2Rs, BitterDB contains information about approx. 1000 molecules that have been reported as bitter in humans, as well as other species (Wiener et al. 2012; Dagan-Wiener et al. 2019). Moreover, the database lists, if available, which TAS2Rs bind these bitter compounds, along with mutations that can affect receptor response and frequently present genetic variants (Wiener et al. 2012; Dagan-Wiener et al. 2019). Furthermore, already precomputed homology models for TAS2Rs are provided, facilitating future structure-based computational studies (Dagan-Wiener et al. 2019; Di Pizio et al. 2020a, b). Ligand-based studies have already been carried out. Chemoinformatics analysis have been applied to the list of bitter compounds in BitterDB to investigate the promiscuity, toxicity or drug-like properties of bitter compounds (Di Pizio and Niv 2015; Nissim et al. 2017; Di Pizio et al. 2020a, b). In addition, machine learning algorithms have been trained to predict bitterness (Dagan-Wiener et al. 2017; Zheng et al. 2018; Banerjee and Preissner 2018; Margulis et al., 2020).

For ORs, the amount of the data is significantly larger (especially when considering not only the ~ 400 human ORs, but also ORs from mouse and other species) and is distributed among several databases (Marenco et al. 2016; Di Pizio et al. 2020a, b). For instance, ORDB (Crastro et al. 2002), OlfactionDB (Modena et al. 2011), OdorDB (Marenco et al. 2013) and the Leibniz-LSB@TUM Odorant Database (Dunkel et al. 2014; Kreissl et al. 2019) contain information about odorant molecules and/or their cognate olfactory receptors. Complementarily, HORDE (Olender et al. 2013) is dedicated to olfactory receptor SNPs and haplotypes and their frequency in the population, whereas hORMdb (Cierco-Jiménez et al. 2021) additionally maps the sequence variants onto known topological positions of class A GPCRs to predict the functional impact of such mutations. As in the case of bitter tastants, machine learning approaches have also been developed for odorants (Lötsch et al. 2019). These algorithms aim at predicting either new ligand-receptor pairs (Liu et al. 2011; Audouze et al. 2014; Bushdid et al. 2018; Caballero-Vidal et al. 2020; Cong et al. 2020) or smells (Keller et al. 2017; Poivet et al. 2018; Nozaki and Nakamoto 2018; Chacko et al. 2020; Sharma et al. 2021), based on chemical features of the odorants.

## Future Directions

The interplay of experimental and computational approaches has enabled a deeper molecular characterization of bitter taste and olfaction. However, the lack of experimental structures of bitter taste and olfactory receptors is still a hurdle that limits the accuracy of the computational structural models that can be generated. Moreover, a more extensive characterization of the physiological and pathological roles of extraoral TAS2Rs and extranasal ORs is required to exploit their potential as novel drug targets. In addition to computational molecular modeling and data science approaches, systems biology is expected to contribute to further understand the connection between ligand-receptor recognition and the subcellular response of the corresponding type II taste cell or olfactory sensory neuron.
